# Antibiotic ampicillin induces immune tolerance in renal transplantation by regulating the proportion of intestinal flora in mice

**DOI:** 10.3389/fcimb.2022.1048076

**Published:** 2022-11-18

**Authors:** Xiaoqiang Wu, Guanghui Cao, Zhiwei Wang, Xuan Wu, Xiangyong Tian, Yue Gu, Fengmin Shao, Tianzhong Yan

**Affiliations:** ^1^ Department of Urology, Henan Provincial Clinical Research Center for Kidney Disease, Henan Provincial People’s Hospital (Zhengzhou University People’s Hospital), Zhengzhou, Henan, China; ^2^ Department of Nephrology, Henan Provincial Clinical Research Center for Kidney Disease, Henan Provincial People's Hospital (Zhengzhou University People's Hospital), Zhengzhou, Henan, China

**Keywords:** renal transplant, intestinal flora, antibiotic, 16S rDNA gene sequencing, renal injury

## Abstract

**Objectives:**

There are significant differences in the composition of intestinal flora in renal transplant recipients before and after an operation, which has a great impact on the prognosis of renal transplantation. The purpose of this project is to study the effect of intestinal flora imbalance on renal transplantation.

**Methods:**

The animal model of renal transplantation was established after intestinal flora imbalance (mice pretreated with compound antibiotics), or the animal model of renal transplantation was established after being pretreated with single antibiotics. HE, PAS, and Masson staining was used to detecting the histopathological changes of transplanted renal. The expression of inflammatory factors and infiltration of inflammatory cells of renal tissue were respectively been detected by ELISA kit and flow cytometry.

**Results:**

Antibiotic pretreatment restored weight loss, and decreased serum creatinine level in mice after renal transplantation. The tissue staining, ELISA assay, and flow cytometry data showed that antibiotic pretreatment alleviated injury of the renal allograft, inhibited the inflammatory factors levels, and reduced inflammatory cell infiltration in mice after renal transplantation. Furthermore, single antibiotic, especially ampicillin pretreatment can also play the same role as compound antibiotics, such as restoring weight loss, decreasing serum creatinine level, alleviating renal allograft injury, inhibiting inflammatory factors levels, and reducing inflammatory cell infiltration in mice after renal transplantation.

**Conclusions:**

Antibiotic ampicillin may inhibit inflammatory cell infiltration after renal transplantation by regulating the proportion of intestinal flora in mice, to reduce renal injury and play a role in renal protection.

## Introduction

The gastrointestinal tract of the human body is rich in hundreds of millions of microbial. The genome encoded by these microbes is 50-100 times that of the human body, and known as the “second genome” of the human body. As a “special organ” of the human body, intestinal flora coexists with the human body and plays an important role in the metabolism of substances and energy, nerve and immune regulation, as well as resisting the invasion of pathogenic microorganisms (Zhou et al., 2020). Numerous studies have shown that dysbacteriosis (changes in the structure of flora) is closely related to the occurrence and development of a variety of diseases, such as cancer (Tilg et al., 2018), infectious diseases (Ghani et al., 2022), cardiovascular diseases (Xu and Yang, 2021), mental diseases (Järbrink-Sehgal and Andreasson, 2020).

Renal transplantation is the first choice for patients with end-stage renal disease. With the use of new immunosuppressants, the short-term prognosis and long-term survival of renal transplant recipients improved (Gioco et al., 2020). However, after kidney transplantation, a large number of inflammatory cells infiltrated the graft. These cells play a role in immune response and inflammatory response after ischemia-reperfusion, resulting in a series of damage to the recipient (Zhao et al., 2018). In previous animal experiments, pretreatment with antibiotics before transplantation can delay the rejection of skin transplantation with major antigen mismatch and heart transplantation with MHC-II molecular mismatch (Lei et al., 2016). Therefore, the composition of intestinal microorganisms may predict the occurrence of rejection. Changing the tissue of intestinal flora by targeting may become one of the strategies to improve the immune tolerance of grafts.

Antibiotics frequently used to treat bacterial infections. Although antibiotics kill bacteria and inhibit their growth, they can also induce drug resistance (Zimmermann and Curtis, 2019). In addition, antibiotic use can temporarily or permanently alter the composition of the intestinal microbiota and promote colonization by intestinal pathogens (Freifeld et al., 2011). In recent years, intestinal flora has become a research hotspot to explore the common complications after renal transplantation from a perspective of intestinal flora. Studies have shown that the composition of intestinal flora in renal transplant recipients is significantly different before and after surgery, which is closely related to the occurrence and development of many complications after renal transplantation and affects the prognosis of recipients (Swarte et al., 2020). Therefore, it is an economic and effective intervention measure to use antibiotic pretreatment to improve the intestinal flora composition and to alleviate the related complications after renal transplantation.

In this study, we first established a renal allograft mouse model after clearing intestinal flora with compound antibiotics, to determine the effect of compound antibiotic pretreatment on renal allograft mice. Then, the model of renal allograft mice was pretreated with a single antibiotic to determine the effect of single antibiotic pretreatment on renal allograft mice. Finally, according to the experimental results of mice, it was determined that the ampicillin played the most significant role, and the 16s RNA gene sequencing technology was used to analyze the effect of ampicillin on the intestinal flora of renal allograft mice. This study aimed to observe the effect of antibiotic pretreatment on inflammatory cell infiltration in mouse renal allografts and to explore the possible mechanisms of antibiotic pretreatment on inflammatory cell infiltration.

## Material and methods

### Animal experiment

Male Balb/c mice were purchased from Shanghai SLAC Laboratory Animal Co.,Ltd, and maintained in a specific pathogen-free room in the Experimental Animal Center at Zhengzhou University, in cages with free access to water and food and a 12-h/12-h light/dark cycle. The mice were organized into 4 groups (Control group, intestinal flora imbalance group (antibiotics, ABx), renal transplantation group (Transplant), intestinal flora imbalance +renal transplantation group (ABx+Transplant), every group consists of 6 mice for a total of 30 mice). Mice in ABx group received broad-spectrum compound antibiotic treatment. The antibiotics were added to the drinking water based on the weight of the mice, Amp (100 mg/kg ampicillin), Van (50 mg/kg vancomycin), Met (100 mg/kg metronidazole), and Neo (100 mg/kg neomycin). The mice received antibiotics for 4 weeks. Mice in ABx+Transplant group were treated with broad-spectrum compound antibiotics (Am, Van, Neo, and Met) for 4 weeks to establish mice with intestinal flora imbalance, and then underwent renal transplantation. The mice were been killed one week after renal transplantation.

### Allogeneic renal transplantation in mice

The mouse model of the renal transplant was established as previously described (Liu et al., 2019). In brief, mice underwent a standard midline abdominal incision under anesthesia with inhalation of 2% isoflurane. Then, the donors’ left kidneys, aorta, inferior vena cava, and ureter were been removed under a microscope followed by lavage *in situ* with the histidine-tryptophane-ketoglutarate solution. The isolated renal were then implanted below the level of native renal vessels of recipients with left nephrectomy, while the infrarenal aorta and the inferior vena cava were perfectly anastomosed to the recipients. In addition, the ureter was been directly anastomosed to the bladder for urinary tract reconstruction.

### Sampling and testing

Blood was collected from mouse orbit and placed at room temperature for 2 hours, then been centrifuged. The supernatant was taken and stored at -20°C. Serum creatinine level was measure by automatic biochemical instrument. Tumor necrosis factor in serum α (TNF-α), interferon-γ (IFN-γ), Interleukin 1β (IL-1β), and Interleukin-6 (IL-6) in renal tissues were determined by enzyme-linked immunosorbent assay (ELISA).

### Staining and histopathology

After taking blood, the mice were been killed, and the renal tissue (transplanted renal tissue) was taken out. The floating blood on the surface of renal tissue was been washed with normal saline, dried with filter paper, and fixed in formalin. Histopathological examination: the tissues were been removed from formalin solution, dehydrated, embedded in paraffin, sectioned, stained with conventional HE, PAS, and Masson, and observed and photographed under the optical microscope.

### Flow cytometry analysis of cell infiltration in renal tissue

The renal tissues were cut into pieces of approximately 2 mm^2^, and digested by tyrosinase plus 0.5% type II collagenase at 37°C with 5% CO_2_ for 2 h and meshed through a 200-gauge stainless steel filter. Cells are collected using centrifugation at 1500 rpm for 10 min at 4°C and resuspended in PBS. The single nucleus cell suspension was been collected and analyzed by flow cytometry. The percentage of monocytes (CD11b^+^LY6C^+^), macrophages (CD11b^+^F4/80^+^), and neutrophils (CD11b^+^LY6G^+^) cells in the mouse renal were determined by flow cytometry. Briefly, cell suspension at 106/tube was stained in duplicate with FITC-anti-CD11b, APC-F4/80, and PE-anti-LY6C/LY6G at room temperature for 30 min in the dark. After washed with PBS, the cells were been analyzed by flow cytometry on the FACSCalibur with FACSDiva software.

### Sequencing analysis of intestinal flora by Illumina MiSeq

The high-throughput sequencing and analysis of the experimental flora structure were completed by Shanghai CapitalBio Technology Co., Ltd. DNA was extracted from the intestinal mucosa of mice according to the instructions of the qubit dsDNA assay kit (life technologies, q328520). The extracted DNA samples were detected by qubit 2.0 fluorometer (Invitrogen, Carlsbad, CA), and the metavxtm library construction kit was used to construct the sequencing library. Using 30 ~ 50 ng DNA as a template, the V3-V4 variable region on bacterial 16S rDNA was amplified by PCR, 341F: 5’-CCTAYGGGRBGCASCAG-3’, 806R: 5’-GGACTACNNGGGTATCTAAT-3’ (Jiang et al., 2020). The quality of the library was been detected by Agilent 2100 biological analyzer, and qubit quantification was performed. After the DNA library is mixed, it is been sequenced on the computer, and the sequence information is read by miseq control software. The two terminal sequences are de hybridized, spliced, and chimeric sequences are been removed. Vsearch is used for sequence clustering (the sequence similarity is set to 97%), and the corresponding species information of each OTU is obtained by referring to the silva132 database. RDP classifier software is used for species comparison annotation, and the reserved confidence interval is greater than 0.7. Taxon clustering, OTUs abundance, and α or β diversity analysis were performed on the measured effective data. Lefse analysis was been used to compare the differences in intestinal microflora abundance in renal transplantation mice caused by antibiotics at the level of species classification.

### Statistical analysis

Data expressed as the mean ± standard deviation (SD). The comparisons among groups were done by one-way analysis of variance (ANOVA), followed by Tukey *post hoc* testing. The *P*< 0.05 was been considered statistically significant.

## Results

### ABx pretreatment improves renal function in renal transplantation mice

**Figure 1:**
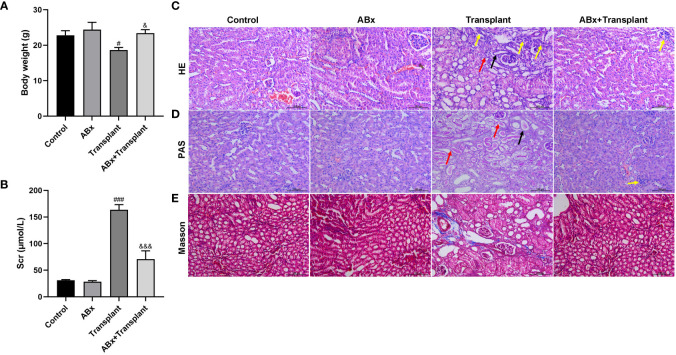
Effect of intestinal flora imbalance on renal transplantation in mice. Male BALB/c mice were purchased and randomly divided into 4 groups: Control group, intestinal flora imbalance group (antibiotics, ABx), renal transplantation group (Transplant), intestinal flora imbalance +renal transplantation group (Abx+Transplant). **(A)** The body weights. **(B)** The serum creatinine level. **(C-E)** HE, PAS and Masson staining for pathological examination. The yellow arrow indicates diffuse inflammatory cell infiltration, the black arrow indicates tubular necrosis, and the red arrow indicates tubulitis. ^#^p < 0.05, ^###^p < 0.001 vs Control group; ^&^p < 0.05, ^&&&^p < 0.001 vs transplant group.

To study the effect of intestinal flora imbalance on renal transplantation in mice, the mice were divided into 4 groups: Control group, intestinal flora imbalance group (antibiotics, ABx), renal transplantation group (Transplant), intestinal flora imbalance +renal transplantation group (ABx+Transplant). Compared with the control group, the weight of mice in the transplantation group decreased significantly and compared with kidney transplantation mice, ABx pretreatment significantly increased the weight of kidney transplantation mice, while only ABx pretreatment did not affect the body weight of mice (**Figure 1A**). Creatinine is a product of muscle metabolism in the body, and mainly discharged from the body through the glomerulus. We can judge whether the kidney is healthy by measuring the value of serum creatinine. As shown in **Figure 1B**, only ABx pretreatment did not affect the serum creatinine level. Compared with the control group, the serum creatinine in the transplant group increased significantly, which decreased in renal transplantation mice that pretreated with ABx. And the (transplanted) renal tissues of mice in each group were stained with H&E and PAS (**Figures 1C, D**). The results showed that the kidneys of ABx pretreated mice were normal, however, the diffuse inflammatory cells infiltrated the renal parenchyma, necrotic tubules and tubulitis can been observed in the transplanted renal tissues of the mice transplant group. At the same time, ABx pretreatment can effectively alleviate renal inflammation in renal transplantation mice. In addition, we also performed Masson staining on the (transplanted) renal tissues of mice in each group. As shown in **Figure 1E**, we observed slight collagen fibers in the renal tissues of mice in the transplant group, and renal tissues of mice in the ABx group and ABx+ transplant group showed normal. These results indicated that ABx pretreatment improved renal function in renal transplantation mice.

### ABx pretreatment inhibits inflammatory cell infiltration in grafts of renal transplantation mice

**Figure 2:**
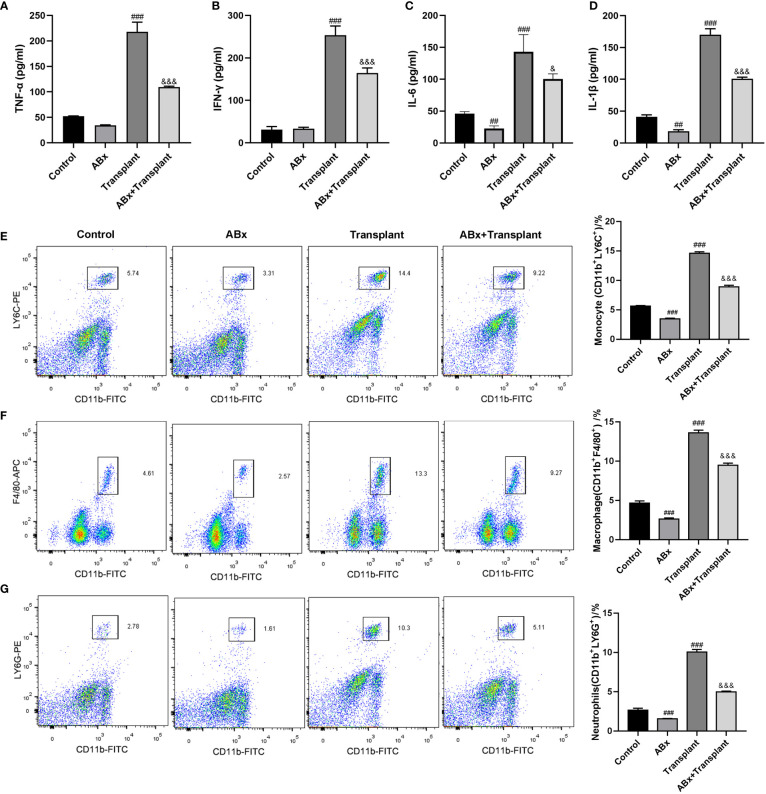
Effect of intestinal flora imbalance on inflammatory response in renal transplantation mice. **(A-D)** ELISA was used to detect the expression levels of TNF-α、IFN-γ、IL-6 and IL-1β in renal tissue. **(E-G)** Flow cytometry used to detect the infiltration of inflammatory cells (monocytes, macrophages, neutrophils) in the renal tissue. ^##^p < 0.01, ^###^p < 0.001 vs Control group; ^&^p < 0.05, ^&&&^p < 0.001 vs transplant group.

To explore the effect of intestinal flora imbalance on inflammatory response in renal transplantation mice, mice grouping was the same as in **Figure 1**. The expression levels of pro-inflammatory and inflammatory (TNF-α、IFN-γ、IL-6, and IL-1β) in (transplanted) renal tissue were detected by ELISA kit. As shown in **Figures 2A-D**, there are high levels of TNF-α、IFN-γ、IL-6 and IL-1β in the transplanted renal tissue of renal transplantation mice. ABx pretreatment can significantly reduce the inflammatory response of renal transplantation mice. Then, we detected the infiltration of inflammatory cells [monocytes (CD11b^+^LY6C^+^), macrophages (CD11b^+^F4/80^+^) and neutrophils (CD11b^+^LY6G^+^)] in the (transplanted) renal tissues of mice in each group by flow cytometry. The results showed that compared with the control group, ABx pretreatment could reduce the inflammatory cell infiltration in the renal tissue of mice in each group, and at the same time, the inflammatory cell infiltration in the transplanted renal tissue of transplantation mice increased significantly. Compared with the renal transplantation group, the inflammatory cell infiltration in the transplanted renal tissue of ABx pretreated renal transplantation mice was significantly reduced (**Figures 2E-G**). These data suggested that ABx pretreatment inhibits inflammatory cell infiltration in grafts of renal transplantation mice.

### Antibiotics ampicillin pretreatment improves renal function in renal transplantation mice

**Figure 3:**
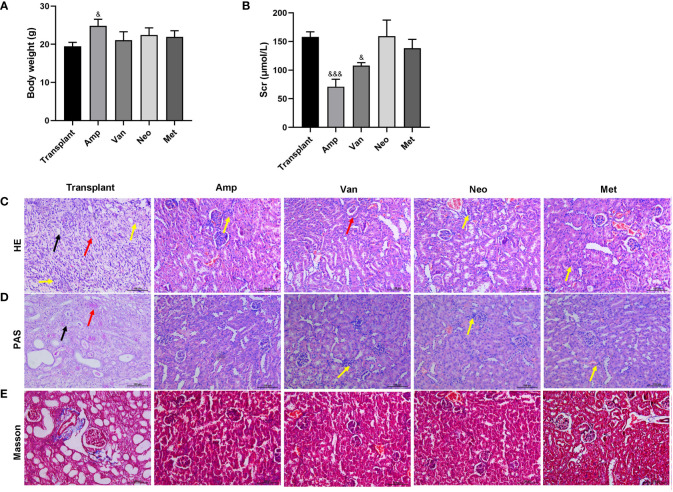
Effect of single antibiotic on renal transplantation in mice. Balb/c mice were treated with antibiotics ampicillin (Amp), vancomycin (Van), neomycin sulfate (Neo), and metronidazole (Met), respectively, and then underwent renal transplantation. **(A)** Compare the body weight of mice in each group. **(B)** The serum creatinine level. **(C-E)** HE, PAS and Masson staining were used to pathological examination of the transplanted renal. The yellow arrow indicates diffuse inflammatory cell infiltration, the black arrow indicates tubular necrosis, and the red arrow indicates tubulitis. ^&^p < 0.05, ^&&&^p < 0.001 vs transplant group.

To determine the effect of a single antibiotic on renal transplantation in mice, the mice were divided into 5 groups: Transplant, ampicillin (Amp), vancomycin (Van), neomycin sulfate (Neo), and metronidazole (Met). Except for the simple transplantation group, the other 4 groups of mice were pretreated with corresponding antibodies for 4 weeks before renal transplantation. Compared with the renal transplantation group, the weight of mice in the amp group increased significantly, while the weight of mice in the other 4 antibiotic pretreatment groups did not change significantly (**Figure 3A**). Compared with the transplantation alone group, the serum creatinine significantly reduced in the Amp and Van pretreatment groups (**Figure 3B**). As shown in **Figures 3C, D**, the HE and PAS stain results showed that diffuse inflammatory cells infiltrated the whole renal parenchyma, necrotic tubules and tubulitis were observed in the transplant group. Compared with the transplant group, the renal tissue inflammation in the other 4 antibiotic pretreatment groups were relieved. Among them, the renal tissue of the Amp pretreatment group was the closest to normal renal tissue, and there was no significant difference among Neo, Met and Van pretreatment groups. Masson staining showed that a small number of collagen fibers could be seen in the transplanted renal tissue of mice in the transplant group, and the renal tissue of mice in the other 4 antibiotic pre-treatment was normal (**Figure 3E**). These results showed that a single antibiotic, especially ampicillin pretreatment, could improve the renal function of renal transplantation mice. These results suggested that antibiotics ampicillin pretreatment improved renal function in renal transplantation mice.

### Antibiotics ampicillin pretreatment inhibits inflammatory cell infiltration in grafts of renal transplantation mice

**Figure 4:**
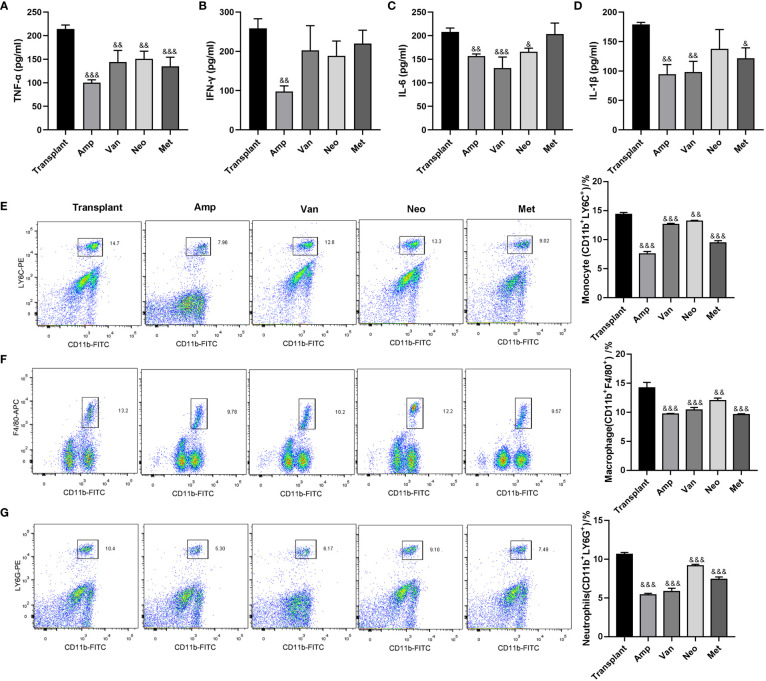
Effect of single antibiotic on inflammatory response in renal transplantation mice. **(A-D)** ELISA was used to detect the expression levels of TNF-α、IFN-γ、IL-6 and IL-1β in the transplanted renal. **(E-G)** Flow cytometry used to detect the infiltration of inflammatory cells (monocytes, macrophages, neutrophils) in the transplanted renal. ^&^p < 0.05, ^&&^p < 0.01, ^&&&^p < 0.001 vs transplant group.

To further explore the effect of a single antibiotic on inflammatory response in renal transplantation mice, mice grouping was the same as **Figure 3**. The expression levels of pro-inflammatory and inflammatory factors (TNF-α、IFN-γ、IL-6 and IL-1β) in transplanted renal tissue of ampicillin-pretreated renal transplantation mice were significantly lower than that in the transplant group. Ampicillin pretreatment significantly reduced the TNF-α, IFN-γ, IL-6 and IL-1β levels, vancomycin pretreatment reduced TNF-α, IL-6 and IL-1β levels, neomycin sulfate pretreatment reduced TNF-α and IL-6 level, and metronidazole pretreatment reduced TNF-α and IL-1β level. Only ampicillin pretreatment could inhibit the expression of all pro-inflammatory and inflammatory factors in transplanted kidney tissue of renal transplantation mice (**Figures 4A-D**). In addition, the flow cytometry results showed that, compared with the renal transplantation group, the inflammatory cell infiltration in the renal tissue of single antibiotic-pretreated renal transplantation mice were all significantly reduced (**Figures 4E-G**). These data suggested that ampicillin pretreatment inhibits inflammatory cell infiltration in grafts of renal transplantation mice.

### Ampicillin pretreatment reduces intestinal microflora diversity in renal transplantation mice

**Figure 5:**
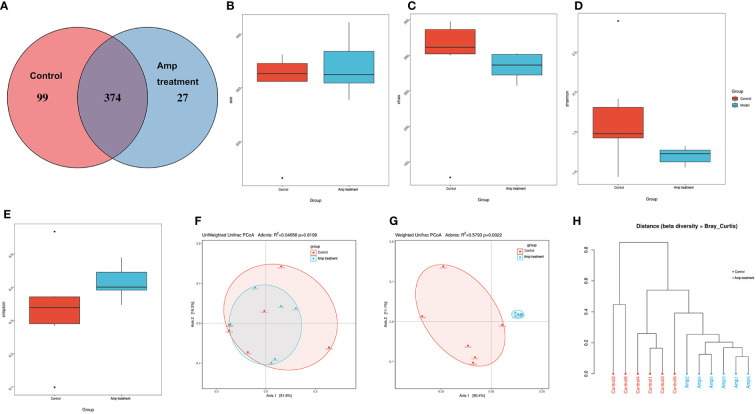
Effect of ampicillin on intestinal microflora diversity in renal transplantation mice. **(A)** OTU Venn diagram. Each note represents a group (n=6), and the number of the overlapping part between the color graphs refers to the total number of OTUs between the two groups, while the non-overlapping part refers to the number of OTUs unique to each group. **(B-E)** Difference analysis of alpha diversity index between the two groups. **(B)** ace, P-value: 0.8182. **(C)** chao, P-value: 0.132. **(D)** Shannon, P-value: 0.132. **(E)** simpson, P-value: 0.065. **(F, G)** Difference analysis of beta diversity index between the two groups using principal coordinate analysis (PCoA). PCoA plots using weighted-UniFrac metric and unweighted-UniFrac metric. **(H)** Tree view of similarity of multiple samples.

According to the above results, single antibiotic pretreatment, especially ampicillin pretreatment, could improve the renal function of renal transplantation mice, the intestinal mucosa tissues of antibiotic ampicillin pretreated renal transplantation mice (Amp, n=6) and renal transplantation mice (Control, n=6) were collected. Then we used 16S rDNA sequencing method to analyze the intestinal flora in the intestinal mucosa tissues of two groups of mice. In the present study, we first obtained the pairs of reads by sequencing 12 samples using 16S rRNA gene high throughput sequencing, the reads were spliced and filtered to generate clean tags. According to the USEARCH software based on 97% sequence similarity, the tags were clustered into OTUs. As shown in **Figure 5A**, a total of 500 OTUs were assigned to the simple renal transplantation group and ampicillin pretreated renal transplantation group, 473 and 401 OTUs were found in the renal transplantation group and ampicillin pretreatment group, respectively, and the number of OUTs shared among the two groups was 374. From the comparison of OTU uniform sequence and related diversity index between the two groups, it can be seen that there is no significant difference in Shannon index (p=0.065), Simpson index (p=0.132), Chao I (p=0.132) and ACE index (p=0.8182) between the two groups (**Figures 5B-E**).

Beta diversity reflects the degree of similarity in species diversity of different sample groups, and the small value of beta diversity indicated that the species of the two groups were similar. When considering the existence of species, the transplantation group and ampicillin pretreatment group had similar microbial species, with unweighted UniFrac distances (R^2^ =0.04058; P = 0.8199). When considering species abundance, we find that there were significant changes in the community structure of bacteria of ampicillin pretreatment renal transplantation mice compared to the renal transplantation group, with weighted UniFrac accommodate (R^2 =^ 0.5793; P =0.0022) (**Figures 5F, G**). Next, we use a tree branch structure to describe and compare the similarity and differences between multiple samples. The multi-sample clustering tree based on Bray Curtis suggested that the structure of mucosal flora in the two groups was significantly different (**Figure 5H**).

### Ampicillin pretreatment affects the species composition at the phylum and genus levels in renal transplantation mice

**Figure 6:**
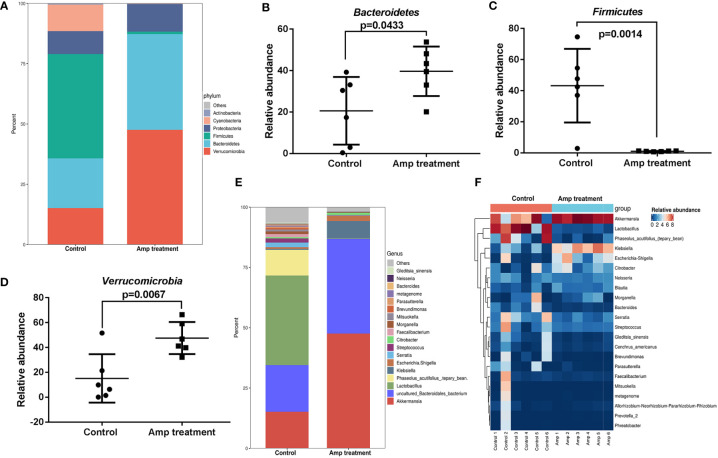
Comparison of species composition of intestinal microflora between the two groups at phylum and genus levels. **(A)** Histograms of intestinal mucosal phylum composition in two groups of mice. **(B-D)** Scatter diagram of three phyla in intestinal mucosa of two groups of mice. **(E)** Histograms of intestinal mucosal genus composition in two groups of mice. **(F)** Heat maps of 20 dominant genus in the two groups.

The two groups of samples included 20 phyla. The four phyla with the highest abundance in the ampicillin pretreatment group were *verrucomicrobia* (47.57%), *Bacteroidetes* (39.63%), *Proteobacteria* (11.45%) and *Firmicutes* (1.03%). In the control group, *verrucomicrobia* accounted for 15.15%, *Bacteroides* accounted for 20.57%, *proteobacteria* accounted for 9.50% and *Firmicutes* accounted for 43.22% (**Figure 6A**). Metastatic analysis showed that there were significant differences in three phylum including *Bacteroidetes*, *Firmicutes*, and *verrucomicrobia* between the two groups (**Figures 6B-D**). Ampicillin pretreatment significantly increased the abundance of *Bacteroides* and *verrucomicrobia* and decreased the abundance of *Firmicutes* at the phylum level.

By comparing with the Silva database, we found 260 genera in 12 samples. Most dominant bacteria in the control group and ampicillin pretreatment group belong to *Firmicutes* and *Proteobacteria* (**Figure 6E**). We plotted the 20 dominant bacterial genera in the two groups into a heat map (**Figure 6F**). Ampicillin pretreatment significantly increased the abundance of *akkermansia*, *Klebsiella*, and *Escherichia-Shigella* at the genus level, and decreased the abundance of *Lactobacillus*.

### Ampicillin pretreatment regulates species abundance in kidney transplantation mice

**Figure 7:**
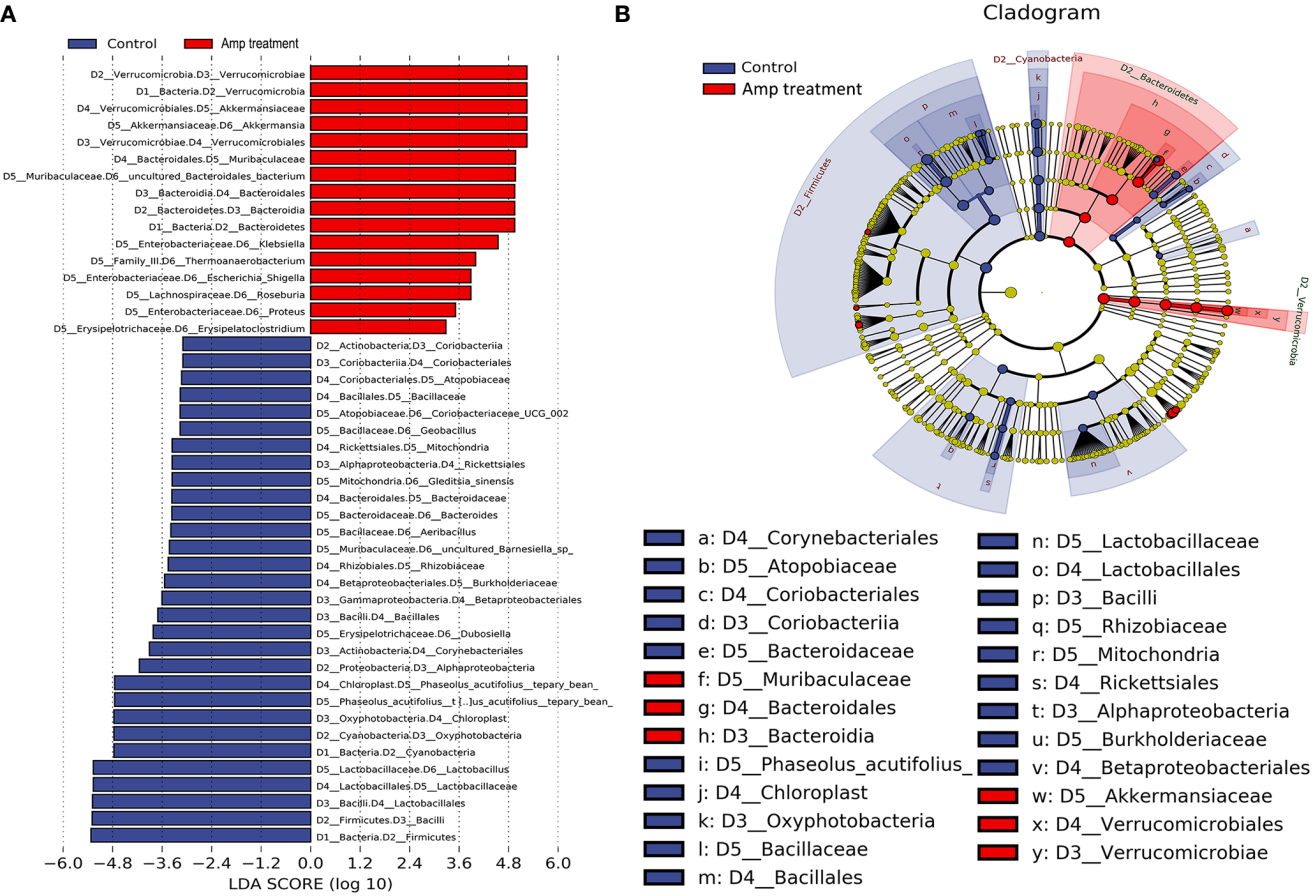
Line discriminant analysis (LDA) effect size (LEfSe) histograms and cladograms between two groups. **(A)** The histogram of LDA value distribution (LDA Score >3) shows the genus with significant differences between control and Amp treatment group. **(B)** The LEfSe cladogram shows the evolutionary relationships between genus with significant differences between thecontrol and Amp treatment group.

To identify high-dimensional biomarkers in the gut microbiota in experiment mice, we performed the line discriminant analysis (LDA) effect size (LEfSe) method. The LDA score was set to 3.0, and different species with an LDA score >3 were considered to be important gut microbiota biomarkers. **Figure 7A** showed the potential microbiota biomarkers at different taxonomic levels in the ampicillin pretreatment group and the most differential biomarkers at the genus level were *Akkermansia*, *Klebsiella*, and *Lactobacillus*. Cladograms of multiple taxonomic level differentiating biomarkers were shown in **Figure 7B**, illustrating bacterial taxa representation between the transplant group and the ampicillin pretreatment group.

## Discussion

Graft injury caused by organ transplantation mainly includes immune rejection and ischemia-reperfusion injury. The injury process is mostly completed by the recipient’s inflammatory cells infiltrating the graft and secreting inflammatory injury factors (Yu et al., 2013). Our results suggested that ampicillin pretreatment may inhibit inflammatory cell infiltration after renal transplantation by regulating the proportion of intestinal flora in mice, to reduce renal injury and play a role in renal protection. The results showed that after antibiotic pretreatment, the expression of inflammatory factors including TNF-α, IFN-γ, IL-6, and IL-1β in the graft of mice undergoing renal transplantation decreased significantly, and the infiltration of inflammatory cells including monocytes, macrophages and neutrophils in the graft decreased significantly, indicating that antibiotic pretreatment can inhibit the infiltration of inflammatory cells in renal transplantation. Furthermore, we found that ampicillin pretreatment could also reduce the expression of inflammatory factors and inflammatory cell infiltration in the grafts.

The intestinal tract is the reservoir of human bacteria, parasitizing more than 1000 kinds of bacteria. Bacterial ectopia induced by mucosal barrier damage and intestinal flora disorder is an important factor leading to infection and systemic inflammatory response. The disorder of intestinal flora not only disturbs the normal functions of intestinal metabolism and nutrition absorption, but also may lead to infection and disturb the normal immune system (Guo et al., 2017). The human gut microbiome is been influenced by many factors, including the host’s genetic factors, dietary habits and drugs. When antibiotics first emerged in the 1940s, it was been called “miracle drugs”. Antibiotics have saved countless lives, but almost all antibiotics cause diarrhea through dysfunction in the gastrointestinal tract, including secretion, digestion, absorption and movement. Antibiotics cause dysregulation of normal intestinal flora, including a substantial reduction in physiological bacteria, and an increase in the reproduction of conditional pathogens (Eyler and Shvets, 2019). Penicillins, sulfonamides, carbapenems, cephalosporins, quinolones, macrolides and aminoglycosides could kill pathogens that cause infection. At this cost, symbiotic bacteria in the human body also suffer “heavy casualties”. Research shows that the antibiotic-sensitive bacteria in patients receiving broad-spectrum antibiotics for a long time are largely been killed, while the insensitive bacteria take the opportunity to reproduce, causing re-infection. And patients are more likely to suffer from obesity, diabetes, asthma, inflammatory bowel disease and other diseases after heavy antibiotic use (Buffie and Pamer, 2013). Antibiotics have saved countless lives, but with the widespread use of antibiotics, people also pay more attention to the side effects of antibiotics (antibiotic-associated diarrhea). In recent years, clinical studies have shown that the main treatment for antibiotic-associated diarrhea is probiotics and fecal microbiota transplantation based on conventional antibiotics (such as vancomycin, metronidazole, etc.). Although probiotics therapy and fecal microbiota transplantation have achieved initial results, the mechanism is still unclear, and there are still potential safety hazards (Mekonnen et al., 2020). Therefore, medical researchers will continue to look for effective alternative treatments.

To clarify antibiotics affect the inflammatory response after renal transplantation by regulating the intestinal flora, we performed a 16S rDNA sequencing analysis on the intestinal mucosal tissues of renal transplantation mice and ampicillin pretreated renal transplantation mice. Principal coordinate analysis and similarity clustering tree showed that there were significant differences between the two groups, suggesting that ampicillin pretreatment significantly affected the intestinal flora. Our results showed that *Verrucomicrobia, Bacteroidetes, Proteobacteria and Firmicutes* were the four most abundant bacterial groups in the intestinal mucosal tissue samples of the control group and amp treatment group. As reported in a previous study, species in *Firmicum* and *Bacteroides* accounted for more than 90% of the total intestinal microbiota (Human Microbiome Project C, 2012). Renal transplantation seems to have significantly changed the composition of the body’s major intestinal microorganisms. Although the impact of changes in intestinal microbial components on solid organ transplantation is still controversial, studies have shown that the bacterial species in these four phyla can affect the side effects of organ transplantation. For example, some bacteria (such as *Clostridium difficile, Enterococcus faecium*, and *Streptococcus*) belonging to *Firmicutes* can infect recipients of solid organ transplantation and are an important cause of side effects (diarrhea, bloodstream infection, pneumonia) after solid organ transplantation in this population (Mullane et al., 2019; Roca-Oporto et al., 2019; Mercuro et al., 2020). Compared with renal transplant recipients without diarrhea, renal transplant recipients with diarrhea have a lower abundance of *Bacteroides* (Lee et al., 2014). Proteus composed of a variety of Gram-negative bacteria (such as *Klebsiella pneumoniae, Pseudomonas aeruginosa and Escherichia coli*) is a risk factor for increased infection, bacteremia and mortality in solid organ transplant recipients (Bodro et al., 2015; Geladari et al., 2017; Ville et al., 2019; Wu et al., 2020). Further analysis of the samples of the two groups at the genus level showed that compared with the control group, the ampicillin pretreatment group has an increased abundance of *akkermansia, Escherichia-Shigella and Klebsiella* in the intestinal tract, while the abundance of *Lactobacillus* decreased significantly. Whether these bacteria with significant changes in expression abundance can cause disease, this needs further study.

Although no research on renal transplantation has reported, whether ampicillin or other antibiotics will change the intestinal flora to induce infection and rejection, and whether the application of probiotics can improve the intestinal flora and clinical prognosis of renal transplantation patients deserve further study, which is also the next direction of our research.

## Data availability statement

The datasets presented in this study can be found in online repositories. The names of the repository/repositories and accession number(s) can be found below: https://www.ncbi.nlm.nih.gov/sra/, PRJNA893084.

## Ethics statement

The animal study was reviewed and approved by Henan Provincial People’s Hospital.

## Author contributions

XiW designed the study and wrote the paper. All authors participated in the experiments and analyzed the data. All authors contributed to the article and approved the submitted version.

## Funding

This work supported by the Henan Provincial Medical Scientific and Technological Research Project (No. LHGJ20200046); the Key Scientific Research Projects in Universities of Henan Province (No.22A320012).

## Conflict of interest

The authors declare that the research was conducted in the absence of any commercial or financial relationships that could be construed as a potential conflict of interest.

## Publisher’s note

All claims expressed in this article are solely those of the authors and do not necessarily represent those of their affiliated organizations, or those of the publisher, the editors and the reviewers. Any product that may be evaluated in this article, or claim that may be made by its manufacturer, is not guaranteed or endorsed by the publisher.
